# Intercropping With Aromatic Plants Increased the Soil Organic Matter Content and Changed the Microbial Community in a Pear Orchard

**DOI:** 10.3389/fmicb.2021.616932

**Published:** 2021-02-12

**Authors:** Yan Zhang, Mingzheng Han, Mengni Song, Ji Tian, Beizhou Song, Yujing Hu, Jie Zhang, Yuncong Yao

**Affiliations:** ^1^Beijing Advanced Innovation Center for Tree Breeding by Molecular Design, Beijing University of Agriculture, Beijing, China; ^2^College of Plant Science and Technology, Beijing University of Agriculture, Beijing, China; ^3^Beijing Key Laboratory for Agricultural Application and New Technique, Beijing, China

**Keywords:** aromatic plants, root exudates, microbial community, soil nutrients, woody-herbaceous intercropping system

## Abstract

Intercropping influences the soil microbiota via litter and root exudate inputs, but the mechanisms by which root exudates mediate the soil microbial community and soil organic matter (SOM) are still unclear. In this study, we selected three aromatic plants (*Ocimum basilicum*, Tr1; *Satureja hortensis*, Tr2; *Ageratum houstonianum*, Tr3) as intercrops that separately grew between rows of pear trees, and no plants were grown as the control in a pear orchard during the spring–summer season for 3 years. The soil from each plot was collected using a stainless-steel corer by five-point sampling between rows of pear trees. The bacterial and fungal communities of the different aromatic intercrops were analyzed by 16S and ITS rRNA gene amplicon sequencing; their functional profiles were predicted by PICRUSt and FUNGuild analyses. The root exudates of the aromatic plants were analyzed by a liquid chromatography-tandem mass spectrometry (LC-MS) system. Compared with the control treatment, all intercropping treatments with aromatic plants significantly increased SOM and soil water content and decreased pH values. The contents of total nitrogen and alkali-hydrolyzable nitrogen in Tr1 and Tr2 were higher than those in Tr3. In Tr3 soil, the relative content of saccharides increased little, whereas the changes in amine (increases) and alcohols (decreases) were rapid. *Ageratum houstonianum* intercropping decreased the microbial community diversity and significantly influenced the relative abundances of the dominant microbiota (Actinobacteria, Verrucomicrobia, Gemmatimonadetes, Cyanobacteria, Ascomycota, and Basidiomycota) at the phylum, class, and order levels, which increased the assemblage of functional groups (nitrite ammonification, nitrate ammonification, and ureolysis groups). Our study suggested that the main root exudates from aromatic plants shaped the microbial diversity, structure, and functional groups related to the N cycle during SOM mineralization and that intercropping with aromatic plants (especially basil and summer savory) increased N release in the orchard soil.

## Introduction

Soil organic matter (SOM) transformation has the dual benefits of improving soil fertility and maintaining the stability of the soil system (Scholes and Noble, 2001). Understanding the mechanisms of SOM transformation by the microbial community is therefore important, as the capacity of soil carbon (C) and nitrogen (N) cycling may be constrained by aboveground plant species (especially invasive plants) and may thereby influence soil nutrient availability (Chen and Stark, 2000; Sardans et al., 2017).

Soil nutrients are generally delivered by plant litter and root exudates, which are slowly released into the soil through microbial processes and SOM mineralization. These processes can alter the degree of nutrient availability through the mineralization of organic compounds (Arancon et al., 2006; Sharma et al., 2011). The functional gene structure of soil microbial communities could influence the decomposition of SOM and consequently regulate the supply of soil nutrients (Gourmelon et al., 2016; Cheng et al., 2017; Waldrop et al., 2017). Many studies have reported that the structure, composition and diversity of the soil microbial community are influenced by plant species, with plant species differences in litter type and root exudates contributing to variation in the physicochemical properties of soil (Gourmelon et al., 2016; Fitzpatrick et al., 2018; Chen et al., 2020). Consequently, these processes drive different modes of ecological functions and services related to nutrient availability in the soil ecosystem (Lekberg et al., 2013).

Fitzpatrick et al. (2018) found that greater similarity in root microbiomes between hosts leads to negative effects on plant performance through soil feedback, with specific microbial taxa in the endosphere and rhizosphere potentially causing competitive interactions among plant species. Reduced crop productivity in field systems repeatedly planted with the same or closely related plant species is a common phenomenon and has been named replanted disease (Mazzola and Manici, 2012). To avoid replanted disease, intercropping is often used in agroecosystems. Interactions between plants and microbes continue to benefit plants by increasing the acquisition of nutrients, producing growth hormones, and defending against enemies (Berendsen et al., 2012). Economically valuable herbs (e.g., forage and flowering ground cover-plants) are often used as intercrops. The influence of plant species and even different genotypes of the same species on the composition of soil microorganisms is considered to be the largest driving factor for soil C and N cycles (Marschner et al., 2005; Koutika et al., 2007; DeHaan et al., 2010). Many studies have shown that intercropping with ground-cover plants can provide valuable organic C accumulation and has positive effects on soil texture and nutrient availability, with feedback regulating plant growth and crop yield (Atucha et al., 2011; Chen et al., 2014). Intercropping with kura clover in the alleys of a pecan orchard increased the content of soil organic carbon (SOC) and the activity of soil enzymes improved the soil structure and reduced soil erosion (Kremer and Kussman, 2011). Ramos et al. (2010) showed that intercropping in almond (*Prunus dulcis* L.) orchards improved soil quality by increasing SOC, soil aggregate stability and microbial activity. The influence of such plant species on soil C accumulation through the regulation of microbial communities has received increasing attention.

Plants used primarily for their aromatic properties in perfumery are defined as aromatic plants in the EU, and they are a source of essential oils, cosmetics and biocides (Lubbe and Verpoorte, 2011; Tang et al., 2013; Song et al., 2014). Intercropping with aromatic plants in an orchard can increase the stability of agroecosystems (Tripathi et al., 2019). Intercropping with certain species of aromatic plants can improve soil quality. For example, intercropping with basil (*Ocimum basilicum* L.) and summer savory (*Satureja hortensis* L.) was found to significantly increase soil organic nitrogen and available nitrogen contents in our previous study (Chen et al., 2014). However, only a limited understanding exists of how this intercropping model directly influences the features of the soil microbial community related to the C and N cycles via root exudates and plant litter and the subsequent impacts on soil nutrient availability.

In this study, to reveal the mechanisms by which root exudates mediate the soil microbial community and regulate SOM transformation and nutrient cycling in an intercroppings system, three species of aromatic plants were selected as intercrops in a pear orchard. The aims were to determine the following: (i) the influences of root exudates from different aromatic plants on soil nutrient cycling; (ii) the effects of these components on the structure, composition, diversity and function of the soil microbial community; (iii) the correlations among root exudates, the soil microbial community and the soil C and N framework in a woody-herbaceous ecosystem; and (iv) the differences among the three intercropping treatments.

## Materials and Methods

### Experimental Site and Design

The field experiment was conducted during the spring-summer season 2008-2010 in an organic pear orchard located in the district of Daxing (39°73′N, 116°33′E), south of Beijing, China. This region is classified as having a temperate, semihumid continental monsoon climate, with a mean annual rainfall of 500 mm and a mean annual temperature of 11.6°C. Approximately 76% of the annual precipitation falls in summer. The orchard, containing 15-year-old *Pyrus pyrifolia* “Huangjin”/*P. betulaefolia* rootstock trees planted at a spacing of 3 × 5 m, has been farmed using organic methods since 2005 and has been managed to meet the organic certification standards set by the Organic Food Development Center of China in 2007. During the natural growth of the pear trees, artificial and physical methods were mainly used to control the pear pests in the whole orchard. The soil type is brown soil according to the classification and codes for Chinese soil (GB/T17296-2009). Before the experimental treatments, the topsoil had a pH of 7.79, SOM content of 10.30 g kg^–1^, alkali-hydrolyzable nitrogen (AN) content of 151.07 mg kg^–1^, available phosphorus (AP) content of 49.16 mg kg^–1^, and available potassium (AK) content of 378.61 mg kg^–1^.

The experiment included four treatments: (i) a control, which was a monocropping system of pear tree (no plants were grown between the rows of pear trees, with naturally occurring weeds removed once a month); (ii) Tr1, an intercropping system of pear tree and basil (*Ocimum basilicum* L); (iii) Tr2, an intercropping system of pear tree and summer savory (*Satureja hortensis* L.); and (iv) Tr3, an intercropping system of pear trees and blue mink (*Ageratum houstonianum* Mill.) (Supplementary Figure S1A). There were 12 plots in total, with three plots in each treatment. Each plot was 5 m wide and 30 m long and included 20 pear trees (Supplementary Figure S1B). The different treatments were separated from adjacent plots by a 10 m isolation belt (Supplementary Figure S1A). The intercrops were sown in a greenhouse, and seedlings (approximately 10 cm in height) were transplanted into the pear orchard in mid-March. The intercrops were grown with a spacing of 0.2 × 0.3 m between the rows of pear trees. During the growing period, intertillage weeding was carried out to keep the intercropping area free of weeds. The aromatic plants in the intercropped plots were mowed in late September annually, with the plant residues left *in situ*.

### Soil Sampling and Processing

Soils from all plots were collected in mid-September 2010 after 3 years of intercropping with aromatic plants (ICAP) or no plants (control). Five soil cores (Supplementary Figure S1C) from each plot were collected using a stainless-steel corer (4.5 cm inner diameter, depth 0–20 cm) and then mixed to obtain a composite sample for each plot. Thus, each treatment had three replicates of soil samples. The composite samples were immediately sieved (<2 mm) and divided into three subsamples, which were immediately stored at −80°C for subsequent DNA extraction and soil microbial biomass carbon (MBC) and soil microbial biomass nitrogen (MBN) determination; stored at 4°C for analyses of enzymatic activity and the soil water content (SWC); and air dried and stored at room temperature prior to chemical analysis.

### Soil Physicochemical Properties and Enzyme Activity Analysis

Soil moisture content was determined gravimetrically by drying at 105°C for 24 h, and the soil pH was determined in a mixture of soil and water suspension (1:2.5) with a pH meter (Mettler Toledo FiveEasy Plus, Switzerland) (Lu, 2000). The SOC was determined using the Walkley-Black method, with slight modifications (Nelson and Sommers, 1996). SOM was calculated as SOC × 1.724 (Pribyl, 2010). Total nitrogen (TN) was determined by the Kjeldahl method (Bremner, 1996), and the total phosphorus was determined by the Olsen method (Olsen and Sommers, 1982). AN was detected using the alkaline hydrolysis diffusion method (Lu, 2000). Soil invertase, catalase (CAT), and urease activities were determined in accordance with the method described by Jin et al. (2009).

The soil MBC and MBN were measured by the fumigation-extraction method (Vance et al., 1987). Briefly, 20 g of fresh soil (dry weight equivalent) was fumigated with CH_3_Cl for 24 h, and then both fumigated and non-fumigated soils were extracted with 75 mL of 0.5 M K_2_SO_4_. The extracted solutions were then filtered and analyzed with a Multi N/C 3100 TOC analyzer (Analytik, Jena, Germany). The soil MBC and MBN were calculated as the difference in extractable organic C and inorganic N contents, respectively, between fumigated and non-fumigated samples using a conversion factor of 0.45 for both C and N (Brookes et al., 1985).

### Soil DNA Extraction and Sequencing

Genomic DNA was extracted from a 0.25 g soil sample using a TIANamp Soil DNA Kit (Tiangen Biotech, Beijing, China) following the manufacturer’s procedures. The quality and quantity of DNA were determined by the A260/280 ratio using a NanoDrop device (NanoDrop 2000, Germany) and electrophoresis (1% agarose gel, including a 1 kb plus ladder). The DNA samples from the soils of the same plot were pooled together and stored at −80°C until PCR amplification. The V3-V4 hypervariable regions of bacterial 16S rRNA was amplified using barcoded primers 341F and 785R (Klindworth et al., 2013), and fungal ITS2 regions (ITS3_KYO2F and ITS4_KYO3R) was targeted for amplification through two rounds of PCR (Toju et al., 2012). The purified PCR amplicons were sequenced using the Illumina MiSeq (300 bp paired-end reads) platform from Ori-Gene Technology Co., Ltd. (Beijing, China) in 2016. The high-quality paired-end reads of the 16S and ITS sequences were merged using FLASH software (Magoč and Salzberg, 2011) and Mothur^1^ to filter the sequences and remove barcodes. The operational taxonomic units (OTUs) were obtained using the UPARSE pipeline based on the merged sequences (Edgar, 2013), and sequences with ≥ 97% similarity were assigned to the same OTU. To obtain the taxonomic information of the OTUs, representative sequences of each OTU were generated and aligned against the SILVA and UNITE databases using the RDP classifier^2^ for the 16S and ITS sequences, respectively (Pruesse et al., 2007). The raw sequences were deposited in NCBI’s Sequence Read Archive (SRA) under BioProject PRJNA685959.

Alpha-diversity indices, including the number of species observed (Sobs) and the Chao and Shannon indices were calculated with Mothur v.1.34.4 (Schloss et al., 2009). The functional profiles of the bacteria were generated from the Functional Annotation of Prokaryotic Taxa (FAPROTAX) database and associated software^3^ (Louca et al., 2016). Trophic classification of pathotrophs, saprotrophs, and symbiotrophs (for fungi) was performed by FUNGuild^4^ with the use of OTUs as described by Nguyen et al. (2016).

### Root Exudate Collection

The rhizosphere soils of six randomly selected aromatic plants in each plot were pooled into a single rhizosphere soil sample. After shaking off the loosely adhering soil, the tightly adhering rhizosphere soil was collected with a brush and passed through a 4 mm sieve. Then, the rhizosphere soils were placed on ice and immediately transported to the laboratory. In the monocropping system (control), we collected the bare soil between the rows of pear trees in the same way as the rhizosphere soil. Ten grams of each soil sample was stored at −80°C for root exudate collection (Jiang et al., 2017). The stored rhizosphere soil samples (200 mg) were thawed on ice, and metabolites were extracted with 1 mL of 50% methanol buffer. Briefly, the mixed solution was vortexed for 1 min, subjected to ultrasound for 20 min (on ice) and centrifuged (4°C, 10000 rpm, 15 min), and then 200 μL of supernatant extraction mixture was stored overnight at −20°C. All samples were analyzed by an AB SCIEX nano LC-MS system (Triple TOF 5600 plus) (SCIEX, United Kingdom) following the manufacturer’s instructions. Root exudate determination was carried out by Sugar Pharma Technology Co., Ltd. (Beijing).

### Statistical Analysis

Soil physicochemical properties, soil enzyme activity, soil microbial community alpha-diversity and composition, and root exudate data were submitted to one-way ANOVA followed by Duncan’s multiple range test. Differences at *P* < 0.05 were regarded as statistically significant. Principal component analysis (PCA) and distance-based redundancy analysis (dbRDA) were used to visualize the associations among soil microbial community parameters, root exudates and environmental variables. Spearman correlation coefficients of pairwise correlations between microbial relative abundance, diversity indicators, soil chemistry, and root exudates were obtained. The statistical analyses and figures were performed using R^5^.

## Results

### Soil Physicochemical Parameters and Enzyme Activities

The soil physicochemical properties in all treatments are summarized in Table 1. SOM and SWC increased, and pH was decreased significantly in the intercropping system compared with the monocropping system (control) values in the pear orchard. The SOM content was increased by 33.6% (Tr1), 34.5% (Tr2), and 35.5% (Tr3) in the intercropping treatments compared with the control. MBC contents increased to a greater extent than MBN content in the intercropping system soil relative to the monocropping system soil values, resulting in an improvement in soil MBC/MBN in the former. The total phosphorus content was increased only in Tr1 and Tr2. Moreover, TN and AN contents and N/P were significantly increased, and C/N was significantly decreased in the intercropping system soil (Tr1, Tr2 and Tr3) relative to the monocropping system soil. TN and AN contents were higher in Tr1 and Tr2 than in Tr3. In addition, the activities of soil enzymes (invertase, urease, and CAT) were significantly enhanced in the intercropping system soil compared with control soil (Table 1).

**TABLE 1:** Basic physicochemical parameters, soil nutrient contents, and enzyme activities in treated and control soil.

Parameter	Control	Tr1	Tr2	Tr3
pH	7.81 ± 0.13a	7.67 ± 0.16b	7.73 ± 0.04b	7.67 ± 0.10b
SWC	8.89 ± 0.90b	11.70 ± 2.37a	10.62 ± 1.56a	10.55 ± 1.47a
SOM	15.21 ± 0.77b	20.33 ± 1.38a	20.46 ± 1.59a	20.62 ± 1.33a
MBC	252.44 ± 20.09b	361.54 ± 37.20a	330.38 ± 59.90a	318.90 ± 47.90a
MBN	73.38 ± 1.17d	88.74 ± 0.97b	94.21 ± 1.27a	85.83 ± 1.44c
MBC/MBN	3.26 ± 0.09c	4.14 ± 0.22a	3.54 ± 0.09b	3.66 ± 0.11b
TN	0.83 ± 0.08c	1.54 ± 0.09ab	1.63 ± 0.05a	1.41 ± 0.05b
TP	0.59 ± 0.02b	0.73 ± 0.04a	0.76 ± 0.03a	0.65 ± 0.04b
AN	99.91 ± 5.79c	133.57 ± 13.72ab	138.16 ± 3.26a	122.79 ± 3.51b
C/N	10.54 ± 0.38a	7.67 ± 0.56bc	7.30 ± 0.85c	8.47 ± 0.32b
N/P	1.23 ± 0.05c	2.24 ± 0.01b	2.35 ± 0.10b	2.42 ± 0.05a
INV	5.08 ± 0.60b	7.24 ± 1.16a	6.75 ± 1.42a	7.16 ± 1.09a
CAT	5.05 ± 0.44b	8.01 ± 0.74a	7.74 ± 1.31a	7.75 ± 1.13a
URE	13.00 ± 1.63b	18.98 ± 3.19a	18.45 ± 3.33a	22.62 ± 3.04a

*Note: Values are the mean ± SD (*n* = 3). Different letters indicate significant differences at *P* < 0.05 based on Duncan’s multiple range tests. SWC, soil water content (%); SOM, soil organic matter (g kg^–1^); MBC, microbial biomass carbon (mg kg^–1^); MBN, microbial biomass nitrogen (mg kg^–1^); TN, total nitrogen (g kg^–1^); TP, total phosphorus (g kg^–1^); AN, alkali-hydrolyzable nitrogen (mg kg^–1^); C/N, soil organic carbon/TN; N/P, TN/TP; INV, invertase (mg glucose g^–1^ soil h^–1^); CAT, catalase (0.1 mol L^–1^ KMnO4 g^–1^ soil h^–1^); URE, urease (mg NH_4_^+^–N g^–1^ soil 24 h^–1^). Control, monocropping system with clean tillage; Tr1, intercropping with basil (*Ocimum basilicum* L.); Tr2, intercropping with summer savory (*Satureja hortensis* L.); Tr3, intercropping with blue mink (*Ageratum houstonianum* Mill.).*

### Root Exudates of Aromatic Plants in Rhizosphere Soil

**FIGURE 1:**
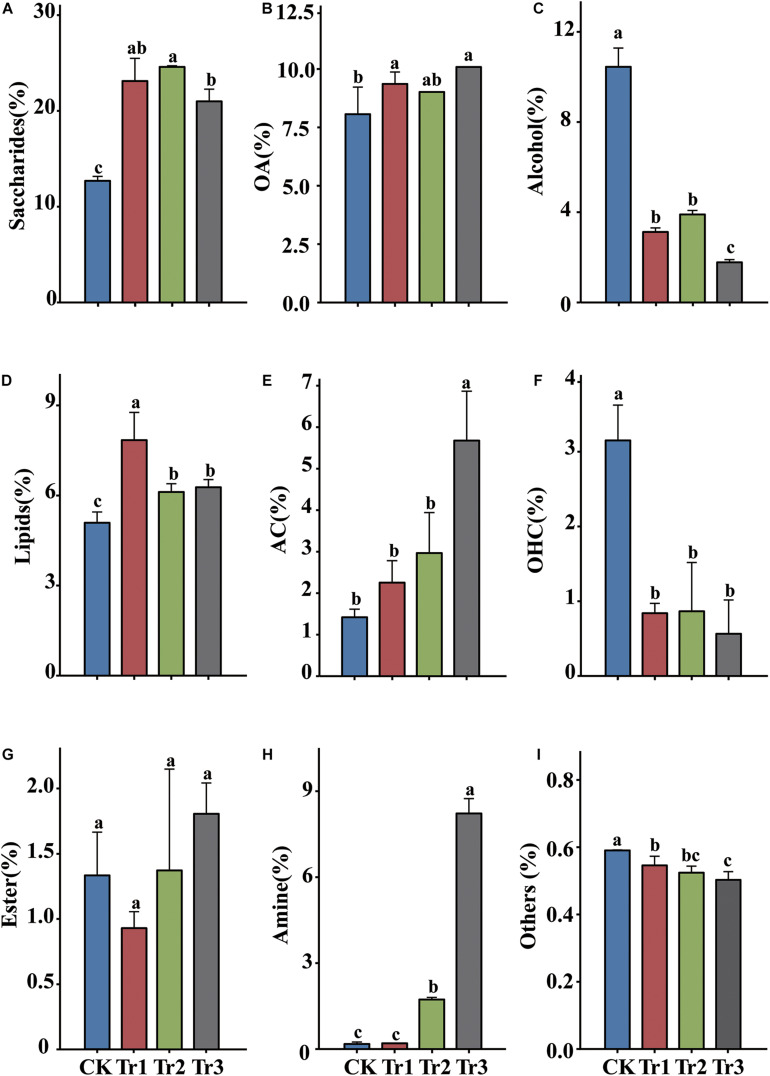
The relative contents of the main root exudate components in rhizosphere soil in intercropping treatments with different species of aromatic plants. **(A)** Saccharides, **(B)** organic acids (OA), **(C)** alcohol, **(D)** lipids, **(E)** aromatic compounds (AC), **(F)** organic heterocyclic compounds (OHC), **(G)** esters, **(H)** amine, and **(I)** others. Values are the mean ± SD (*n* = 3). Different letters indicate significant differences at *P* < 0.05 based on Duncan’s multiple range test.

Metabolomics analysis of the relative contents of the root exudates of aromatic plants revealed that the exudates consisted predominantly of saccharides (up to 24.6%), alcohols (up to 10.5%), organic acids (OA, up to 10.0%), lipids (up to 7.8%), and aromatic compounds (AC, up to 5.7%) (Figure 1). Root exudation was strongly influenced by the intercropping species. In the intercropping systems, compared with the control, the saccharide, OA and lipid contents were increased, but the alcohol and organic heterocyclic compound (OHC) contents were reduced significantly. The alcohol and OHC contents were highest in the control, and the amine and AC contents were highest in Tr3 (Figure 1).

**FIGURE 2:**
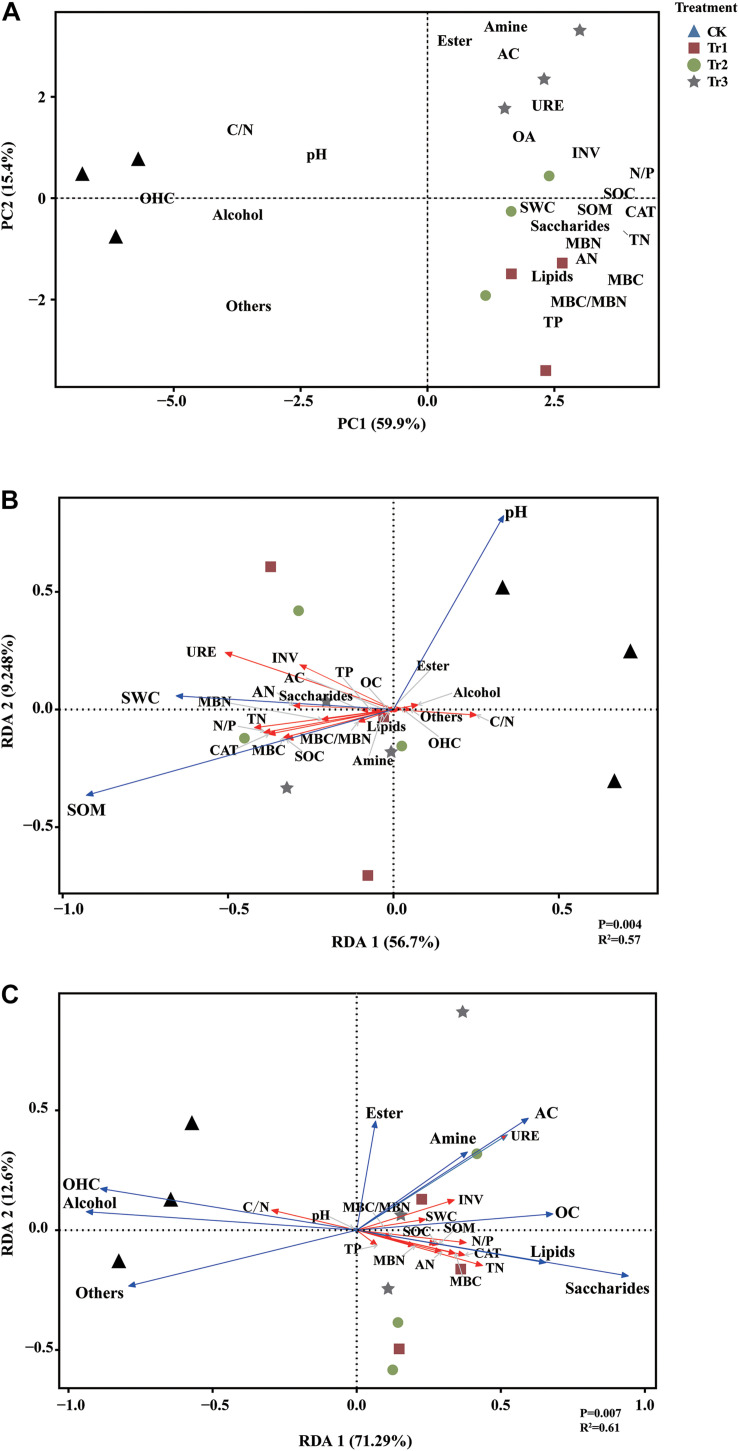
Principal component analysis (PCA) based on the root exudates, soil physicochemical parameters and soil nutrients **(A)** and redundancy analysis (RDA) of soil nutrient variables constrained by basic physicochemical parameters (pH, SWC, and SOM) **(B)** and by root exudates **(C)**.

The PCA results revealed that the significant differences between the intercropping and monocropping systems were associated with PC1, and that the significant differences among the three aromatic plant intercropping systems were associated with PC2. The PC1 and PC2 components accounted for 59.9% and 15.4% of the total variation, respectively (Figure 2A). The C/N ratio, pH, alcohol and OHC contents were significantly positively correlated with each other, while the SWC, SOM, contents of root exudates (AC, saccharides, OA, lipids, amine), contents of C and N compounds, and enzyme activities displayed significant positive correlations. The RDA results indicated that soil pH was positively associated with the contents of root exudates (i.e., alcohols, esters, OHC) and was negatively correlated with the contents of soil C and N compounds and enzyme activities. In addition, SWC was associated with the contents of N compounds, saccharides, OA, and AC, and SOM was associated with the contents of C compounds, saccharides, lipids and amine compounds and CAT activities in the intercropping soil (Figure 2B). The contents of root exudates (saccharides, lipids, OA, AC, amine) were positively related to most soil physicochemical parameters (including soil C and N compounds and the activities of associated enzymes) and negatively correlated with soil pH and the C/N ratio; however, alcohol and OHC contents were positively associated with pH and the C/N ratio (Figure 2C).

### Composition, Structure, and Diversity of the Soil Bacterial Community

**FIGURE 3:**
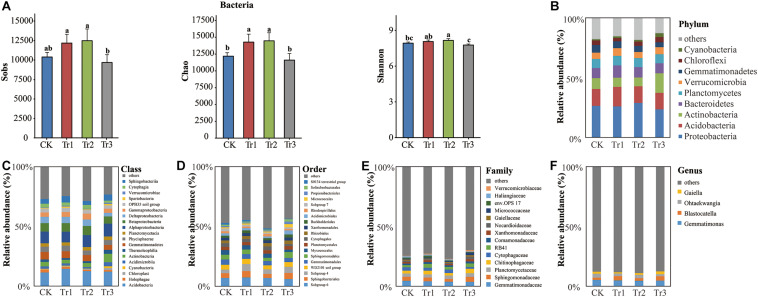
Sobs, Chao, and Shannon indices and phylum-genus composition of the bacterial communities in soil with different species of intercropped aromatic plants, based on the OTUs obtained from 16S rRNA sequencing. **(A)** Sobs, Chao, and Shannon indices; **(B)** phylum; **(C)** class; **(D)** order; **(E)** family; and **(F)** genus. Values are the mean ± SD (*n* = 3). Different letters indicate significant differences (*P* < 0.05) based on Duncan’s multiple range test. Sobs indicates the number of observed species or operational taxonomic units (OTUs). Chao indicates the non-parametric estimation of asymptotic species richness. Shannon’s index was used to assess community diversity.

We sequenced the V3-V4 region of the 16S rRNA gene and obtained a total of 1,968,069 sequences from 12 soil samples (*n* = 3) after filtering. The number of reads ranged from 108,994 to 204,788, with a mean read count of 164,006 ± 28,453. Rarefaction curves implied that the sequencing coverage was sufficient as plateaus were reached for soil communities. Based on the total bacterial community estimated as the number of OTUs, the species richness (Chao index) and alpha-diversity (Shannon index) were enhanced significantly in the intercropping systems with basil and summer savory (Tr1 and Tr2) but not in the intercropping system with plants of blue mink plants (Tr3) compared with the control (Figure 3A).

In all intercropping treatments, nine phyla were dominant: Proteobacteria (26.28%), Acidobacteria (14.56%), Actinobacteria (10.14%), Bacteroidetes (8.96%), Planctomycetes (7.63%), Verrucomicrobia (5.70%), Gemmatimonadetes (5.31%), Chloroflexi (3.64%), and Cyanobacteria (1.76%) (Figure 3B). The intercropping effect of Tr3 on bacterial community composition was more significant than those of Tr1 or Tr2. Intercropping with blue mink (Tr3) significantly increased the relative abundances (RAs) of Actinobacteria and its dominant classes, orders and families, especially Micrococcales and Micrococcaceae, which both increased in abundance to levels approximately three times those of the control. However, there was no significant difference in bacterial community composition between the intercropping treatments with basil and summer savory (Tr1 and Tr2) and the control. Tr3 also significantly increased the RAs of the phylum Verrucomicrobia and its member classes, orders and families and the RAs of the phylum Cyanobacteria and its member classes. Furthermore, Tr3 significantly reduced the RAs of the phylum Gemmatimonadetes and its member classes and genera and those of the class Cytophagia, order Cytophagales, family Cytophagaceae, and genus *Ohtaekwangia* in the phylum Bacteroidetes. Tr1 significantly increased the RAs of the phylum Verrucomicrobia and its dominant classes, orders and families and those of the phylum Bacteroidetes and its class Sphingobacteriia and order Sphingobacteriales. Tr2 significantly reduced the RAs of the phylum Gemmatimonadetes and its member classes but increased the RA of Gammaproteobacteria. In all of the ICAP treatments, the RA of Holophagae in the phylum Acidobacteria was reduced relative to that in the control by 36.7% (Tr1), 54.6% (Tr2), and 43.3% (Tr3) (Figures 3B–F, Supplementary Figure S2A and Supplementary Table S1).

**FIGURE 4:**
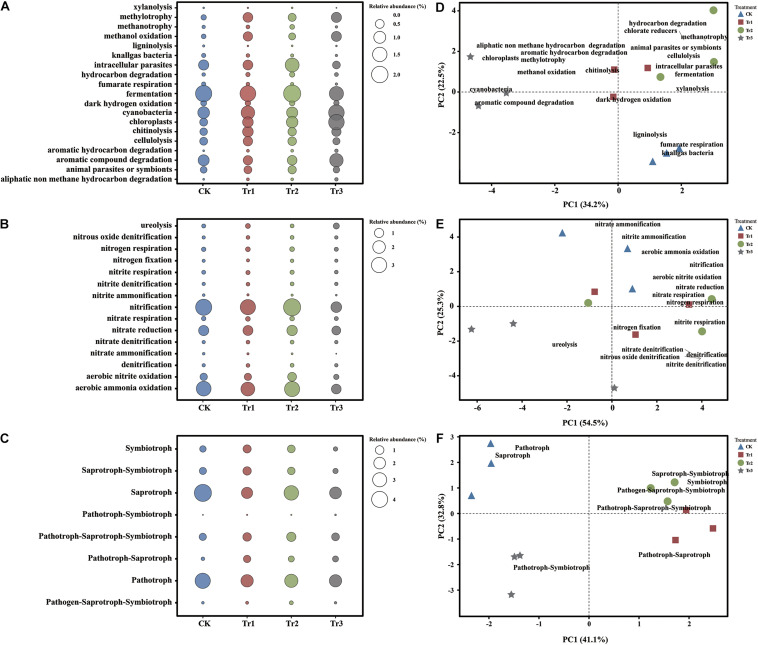
Functional groups in soil with different species of intercropped aromatic plants, identified based on the OTUs obtained from 16S rRNA sequencing. Panels **(A)**, **(B)**, and **(C)** show the changes in functional groups related to the C and N cycles of the bacterial community and the trophic pattern of the fungal community. Panels **(D)**, **(E)**, and **(F)** show the functional group structure related to the C and N cycles of the bacterial community and the trophic pattern of the fungal community.

Based on the FAPROTAX database, we observed that several functions related to the C cycle were significantly altered by ICAP (Figures 4A,D). Compared with the control, all intercropping treatments significantly reduced the assemblage of fumarate respiration, knallgas bacteria, and ligninolysis groups. In addition, Tr3 promoted the assemblage of cyanobacteria, methylotrophy, and methanol oxidation groups, and Tr1 increased the assemblage of cyanobacteria, chitinolysis, and cellulolysis groups (Figures 4A,D). In addition, the assemblage of AC degradation groups showed the largest RA in Tr3 (Figure 4A). With respect to the assemblage of functional groups related to N cycling, the nitrite ammonification and nitrate ammonification groups were reduced significantly by ICAP compared with the control, and they were reduced more significantly in Tr3 than in Tr1 and Tr2. The nitrification and aerobic ammonia oxidation groups were reduced, whereas the ureolysis groups were significantly promoted only in Tr3 (Figures 4B,E). The type of intercropping impacted the structure of the functional groups related to C cycling (Figure 4D) more significantly than that of the functional groups related to N cycling (Figure 4E).

### Composition, Structure, and Diversity of the Soil Fungal Community

**FIGURE 5:**
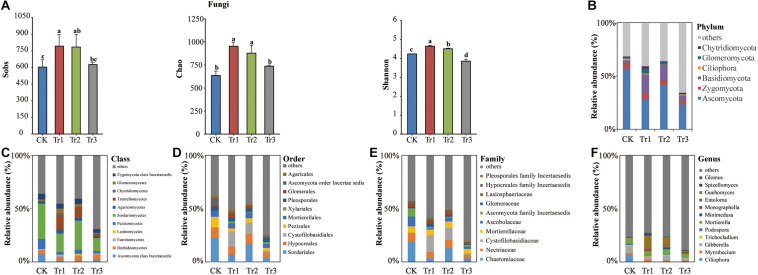
The Sobs, Chao, and Shannon indices and phylum-genus composition of the fungal communities in soil with different species of intercropped aromatic plants, based on the OTUs obtained from ITS rRNA sequencing. **(A)** Sobs, Chao, and Shannon indices; **(B)** phylum; **(C)** class, **(D)** order; **(E)** family; and **(F)** genus. Values are the mean ± SD (*n* = 3). Different letters indicate significant differences (*P* < 0.05) based on Duncan’s multiple range test. Sobs indicates the number of observed species or operational taxonomic units (OTUs). Chao indicates the non-parametric estimation of asymptotic species richness. Shannon’s index was used to assess community diversity.

A total of 359,913 quality-filtered fungal ITS2 sequences were obtained from 12 soil samples (*n* = 3). The number of reads ranged from 16,663 to 36,167 with a mean read count of 29,993 ± 5244. Rarefaction curves implied that the sequencing coverage was sufficient as plateaus were reached for soil communities. Based on the total fungal community estimated as the OTUs, the Sobs, species richness (Chao index) and alpha-diversity (Shannon index) were enhanced significantly by Tr1 and Tr2, while the Shannon index was reduced by Tr3 compared with the control (Figure 5A).

The composition of the total fungal community was dominated by the following phyla: Ascomycota (37.95%), Basidiomycota (9.75%), Zygomycota (4.63%), Glomeromycota (2.20%), Chytridiomycota (1.15%), and Ciliophora (0.96%) (Figure 5B). The RAs of the phylum Ascomycota and its five genera (*Ciliophora, Myrothecium, Gibberella, Trichocladium*, and *Podospora*) were all significantly reduced by ICAP relative to the control. The RAs of class Sordariomycetes, order Sordariales, family Chaetomiaceae, and genus *Mortierella* of Zygomycota were reduced in Tr3 relative to the control, whereas the RA of order Xylariales was promoted significantly by Tr3. Tr1 also influenced the RAs of these taxa and significantly increased the RAs of Basidiomycota and its member taxa Tremellomycetes, Cystofilobasidiales, Cystofilobasidiaceae, and *Guehomyces*; the RAs of Chytridiomycota and its member class Chytridiomycetes; and the RA of *Glomus* of Glomeromycota (Figures 5B–F, Supplementary Figure S2B and Supplementary Table S2).

Based on the FUNGuild database, the analyzed OTUs were assigned to the following guilds: saprotroph, pathotroph, pathotroph-saprotroph-symbiotroph, saprotroph-symbiotroph, symbiotroph, pathotroph-saprotroph, pathogen-saprotroph-symbiotroph, and pathotroph-symbiotroph (Figure 4C). Compared with those in the control, the pathotrophic and saprotrophic groups in ICAP were both reduced significantly. The symbiotic group was promoted by Tr1 and Tr2 but reduced by Tr3 (Figure 4C). The structure of the fungal functional groups related to trophic patterns indicated a clear separation among the four treatments. The pathotrophic and saprotrophic groups were mainly clustered in the control (Figure 4F).

### Relationships Among Soil Properties, Root Exudates, and Microbial Communities

**FIGURE 6:**
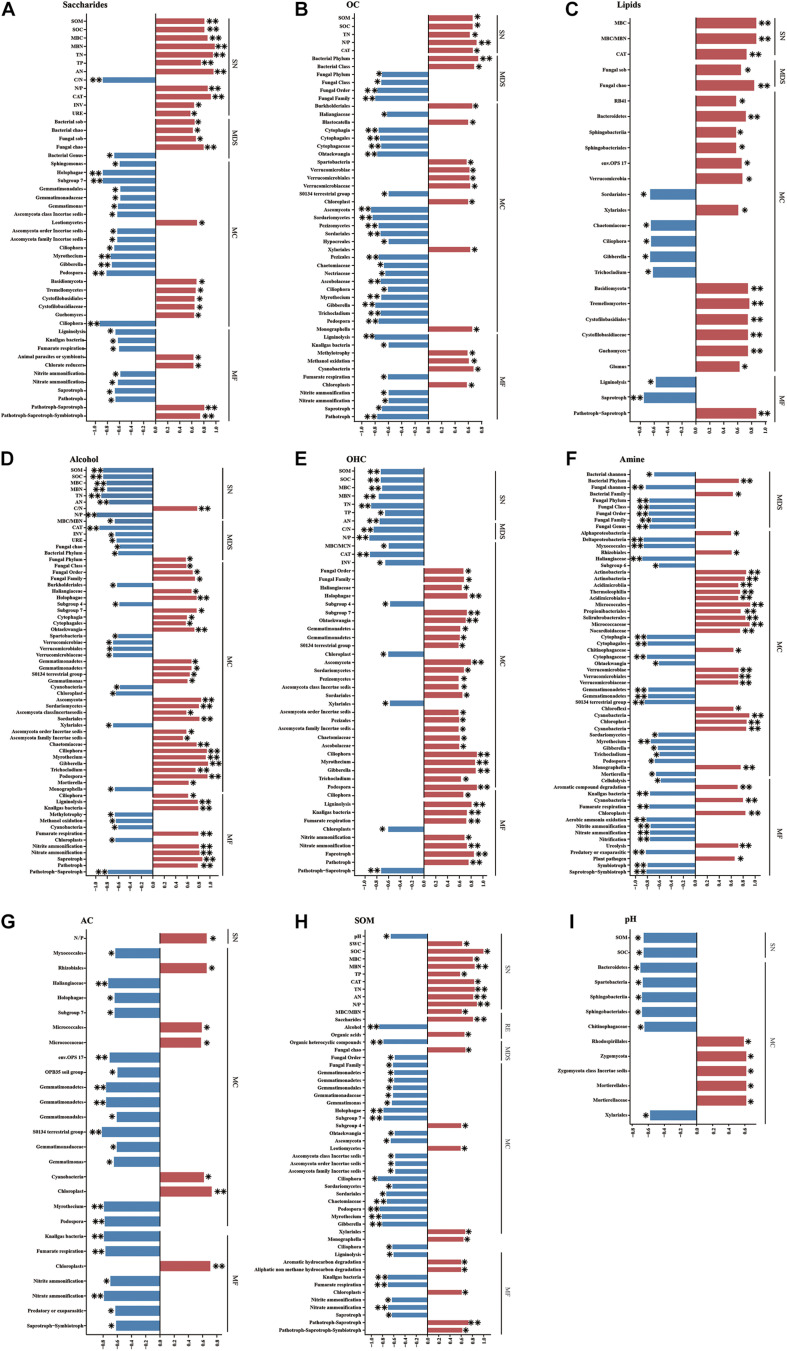
Spearman’s correlation coefficients among soil basic physicochemical parameters, root exudates, microbial communities, and soil nutrients. **(A)** Saccharides and other variables, **(B)** OA and other variables, **(C)** lipids and other variables, **(D)** alcohol and other variables, **(E)** OHC and other variables, **(F)** amine and other variables, **(G)** AC and other variables, **(H)** SOM and other variables, **(I)** pH and other variables. SN, soil nutrients; RE, root exudates; MDS, the diversity and structure of the total microbial community; MC, the composition of the microbial community; MF, functional groups of the microbial community. *P < 0.05, **P < 0.01.

Analyses of the correlations of root exudates and soil properties revealed that saccharide and OA contents were positively correlated with SOM, TN content and CAT activity, whereas alcohol and OHC contents were negatively correlated with these parameters (Figures 6A–E). Regarding microbial diversity, saccharide content was positively correlated with the Sobs and Chao indices of both bacteria and fungi, whereas lipid content was positively correlated with these indices only in fungi. The alcohol content was negatively correlated with the Chao index of fungi, and the amine content was negatively correlated with the Shannon index values of both bacteria and fungi (Figures 6A–G). The contents of seven main root exudates (in this study) were associated with the RAs of the dominant soil microbes. For example, saccharide content was mainly positively correlated with the RAs of Basidiomycota and half of its member taxa and negatively correlated with the RAs of Gemmatimonadales and its member families and genera and some taxa of Ascomycota (though it was positively correlated with Leotiomycetes RA) (Figure 6A). OA content was positively correlated with the RAs of Verrucomicrobiae and its orders and families and negatively correlated with those of Cytophagia and its corresponding orders and families, and most taxa in Ascomycota (Figure 6B). With respect to the functional groups of microbial communities, both saccharide content and OA content correlated negatively with the RAs of ligninolysis, knallgas bacteria, and fumarate respiration groups related to the C cycle; nitrite ammonification and nitrate ammonification groups related to the N cycle; and saprotrophic and pathotrophic groups in the fungal trophic structure. Interestingly, the relationships between alcohols and the RAs of the taxonomic groups and functional groups of dominant microbes were largely opposite those between OA content and the RAs of these groups. In addition, the amine content was positively correlated with the RA of a functional group involved in ureolysis and related to the N cycle (Figure 6D). The AC content was significantly positively correlated with the RAs of Micrococcales and Micrococcaceae (Figure 6G).

Soil organic matter was positively correlated with the contents of OA and saccharides and negatively correlated with the contents of OHC and alcohols in root exudates (Figure 6H). In addition, SOM was positively related to fungal alpha-diversity (Chao). With respect to the RAs of members of the soil microbial community, SOM was mainly negatively correlated with the RAs of Gemmatimonadetes and its member classes, orders, families and genera and most dominant species of Ascomycota, whereas it was only positively correlated with the RAs of the subgroup 4 of order in Acidobacteria and Leotiomycetes of Ascomycota (Figure 6H). Furthermore, SOM was positively correlated with the RAs of some functional groups, such as those involved in aromatic hydrocarbon degradation and aliphatic non-methane hydrocarbon degradation in the bacterial community, and was negatively correlated with the RAs of others, such as saprotrophic groups of the fungal community and the ligninolysis, fumarate respiration, nitrite ammonification, and nitrate ammonification groups of the bacterial community (Figure 6H).

Soil pH was positively correlated with the RAs of Rhodospirillales and Zygomycota (including Mortierellales and Mortierellaceae) and negatively correlated with the RAs of Bacteroidetes (including Sphingobacteriia, Sphingobacteriales, and Chitinophagaceae) and Spartobacteria in Verrucomicrobia, as evaluated by genomics analysis (Figure 6I).

## Discussion

### Root Exudates Shape the Soil C and N Framework After Intercropping

Problems with replanting fruit trees (replant disease) have been reported in numerous fruit-growing areas of the world. In tree fruit production, replant problems have been consistently used to describe conditions of poor tree growth resulting from a plethora of potential factors, including diminished soil fertility, degraded soil structure, and residual herbicide activity (Browne et al., 2006). Culture management should emphasize on improving soil microbial and faunal diversity as well as habitat quality rather than focusing on soil disinfection (Winkelmann et al., 2019). Intercropping is often used in orchards of China because it can mitigate disease and increase fruit yield. Intercropping with Tagetes, conventionally used against nematodes, revealed increased growth of apple in two apple replant disease soils, both in a biotest and in field trials (Yim et al., 2017). Plant diversity in experimental systems often enhances ecosystem productivity, but the mechanisms causing this overyielding are only partly understood (Li et al., 2016). In this study, the root system of intercrops (aromatic plants) is mainly distributed at a depth of 0–20 cm, while that of pear trees are mainly distributed at a depth of 20–60 cm. Thus, there is a complementarity between the root distribution depths of the species. Intercropping, the simultaneous cultivation of multiple crop species in a single field, increases aboveground productivity by enhancing soil C and N due to species complementarity (Cong et al., 2015). High C and N stocks in the soil are correlated with soil productivity (Latati et al., 2017).

In this study, ICAP significantly increased the SOM content and promoted the soil enzyme activities (Table 1). These alterations may have been caused by litter and root exudate inputs from the aromatic plants. Previous studies showed that the total amount of soil C inputs in the undergraded grassland (with high aboveground biomass and living root biomass) was higher than that in degraded grassland due to long-term overgrazing, and root turnover was suggested to be the primary source of soil C inputs (Shen et al., 2020). In our study, the saccharide and OA contents increased while the alcohol and OHC contents decreased after ICAP compared with monocropping. Interestingly, we also found that saccharide and OA contents were positively correlated with SOM, whereas alcohol and OHC contents were negatively correlated with SOM (Figures 6A–E). These results indicated that the changes in root exudates were beneficial for increasing SOM after ICAP. Similarly, a previous study found that SOM can be mobilized by different root exudates (Nardi et al., 2000). Intercropping can contribute to multiple agroecosystem services by increasing yield and improving soil quality and soil C sequestration (Cong et al., 2015). In addition, the input of litter from aromatic plants may have been responsible for the increase in SOM content under intercropping. Castellano et al. (2015) suggested that plant litter was the primary source of all SOM. However, the processes of litter decomposition and SOM stabilization are often considered separate (Sollins et al., 2007). Carrington et al. (2012) found that plant litter quantity rather than quality is the main determinant of the amount of physicochemically stabilized SOM.

The soil nutrient framework was distinct between the intercropping treatments and the control (Figure 2A). Furthermore, the use of different species in the intercropping system resulted in significant differences in the N cycle, with higher TN, AN, and MBN contents and a lower C/N ratio in the soil associated with basil and summer savory plants (Tr1 and Tr2) than in that associated with blue mink plants (Tr3) in the intercropping systems. These differences might be caused by variations in the quantity and quality of plant litter and root exudates among intercrops. High-quality plant litters with high N concentrations and low C/N ratios, accelerated greater mineralization efficiency than low-quality litters (Castellano et al., 2015). In addition, plant root exudates can profoundly modify soil microbial communities and influence their N transformations (Coskun et al., 2017; Meier et al., 2017).

The amount and composition of root exudates varies with the taxonomic status of the host (Chaparro et al., 2014; Mönchgesang et al., 2016; Sasse et al., 2018). In our study, the relative content of AC varied among the different ICAP treatments, with the highest value observed in the Tr3 treatment. One reason for this finding is that among the intercrops, *Ageratum houstonianum* (Tr3) had the highest aboveground and root biomass per plant (data not shown). Previous studies showed that higher plant biomass usually induced higher SOM fraction contents (e.g., particulate organic matter, rhizodeposition), which could act as major sources and energy for microorganisms (Tian et al., 2013).

### Root Exudates From Different Aromatic Plants Contributed to the Distinct Properties of Microbial Community

Some studies have shown that the traits of the soil microbial community are influenced by root exudates (Shi et al., 2011; Hu et al., 2018; Sasse et al., 2018). Additionally, root exudate diversity has been identified as a crucial link between plant diversity and soil microorganisms (Steinauer et al., 2016). We found that intercropping with basil and summer savory (Tr1 and Tr2) improved the species richness and alpha-diversity (Chao index) of the bacterial and fungal communities, whereas no significant difference in these measures was observed between the intercropping treatment with blue mink (Tr3) and the control (Figures 3, 5). These differences might be associated with the higher SOM content and lower pH induced by different aromatic plants. A long-term grassland biodiversity experiment showed that bacterial and fungal diversity increased with higher plant diversity (Lange et al., 2015). Another study showed that intercropping increased microbial OTU richness and fungal community diversity and that the diversity of the soil bacterial community varied among seven intercropping systems (Li and Wu, 2018). These observations show that the intercropping species affects the soil microbial community.

The soil bacterial community displayed two distinct clusters according to the two taxonomic families of the intercrops after ICAP (Supplementary Figure S2A). This finding suggested that the difference in bacterial composition due to interfamily intercropping was larger than that due to intrafamily intercropping. The root exudates of plant species significantly shaped the structure of the rhizosphere bacterial community (Haichar et al., 2008; Sasse et al., 2018). The potential of cropping systems and species identity to modify soil bacterial communities, subsequently modify plant growth and crop-weed competition (Ishaq et al., 2017).

The composition of bacteria was influenced more markedly by blue mink (Tr3) than by basil and summer savory (Tr1 and Tr2) (Supplementary Figure S2A). This might have been due to the contents of aromatic AC, OA and amine being highest in the blue mink (Tr3) rhizosphere. A previous study also indicated that OA in root exudates played a significant role in shaping soil bacterial communities (Shi et al., 2011). Furthermore, SOC and AN have been identified as the key factors shaping bacterial communities in the rhizosphere (Cui et al., 2018). In our study, intercropping with blue mink (Tr3) significantly increased the AC content (Figure 1E), the RAs of the phylum Actinobacteria and its order (Micrococcales) and family (Micrococcaceae) (Figures 3B,D,E). Correlation analysis further revealed that the AC content was significantly correlated with Micrococcales and Micrococcaceae (Figure 6G). We suspected that these groups are involved in the degradation of AC. A previous study found strains resistant to heavy metals in nine of the 15 families belonging to the order Micrococcales (Alvarez et al., 2017). With respect to the fungal community, all ICAP treatments significantly reduced the RAs of Ascomycota and some of its dominant genera, and the effect of intercropping with blue mink (Tr3) was more significant than that of intercropping with basil and summer savory (Tr1 and Tr2) (Figure 5B and Supplementary Figure S2B).

The different aromatic plants induced distinct effects on the functional groups of bacteria involved in soil C and N cycling (Figure 4). In terms of the functional groups related to C cycling, all ICAP treatments significantly recruited the cyanobacteria, the RA of cyanobacteria was increased significantly in Tr3 (Figures 4A,D). Cyanobacteria form the most ancient group of biocrusts and contribute to soil C and N cycling (DeBruyn et al., 2011; Chamizo et al., 2012; Zhao et al., 2015; Muñoz-Rojas et al., 2018; Ma et al., 2019). The three intercropping treatments reduced the assemblage of nitrite ammonification and nitrate ammonification groups, and intercropping with blue mink (Tr3) significantly promoted the assemblage of the ureolysis group (Figure 4B). In addition, intercropping significantly reduced the saprotrophic and pathotrophic groups of fungal trophic types; such reductions might reduce plant disease. Intercropping has been widely used for its beneficial effects in controlling disease and improving nutrition in the field (Lian et al., 2019; Misra et al., 2019).

### Relationships Among SOM, Root Exudates, and Microbial Communities

Soil bacterial and fungal richness (Sobs and Chao indices) was positively correlated with saccharide content (Figure 6A), whereas the alpha-diversity (Shannon index) was negatively correlated with the content of amine compounds (Figure 6F). These results indicated that saccharide compounds increased the richness of the microbial population while amine compounds inhibited the diversity of the microbial community. A previous study found that OA, amine and alcohol compounds were associated with microbial structure (Zhalnina et al., 2018). These observations suggest that root exudates can stimulate microbes to decompose SOM and release N with litter input (Strickland et al., 2015; Meier et al., 2017). The relationships between root exudates and the dominant microbial and functional communities identified based on Spearman analysis differed among the treatments (Figure 6). Therefore, different aromatic plants recruit different dominant microbes in intercropping systems; for example, Tr3 significantly increased the RA of Actinobacteria. Yuan et al. (2015) found that banana root exudates, especially OA, played a crucial role in attracting plant-growth promoting rhizobacteria (PGPR) and initiating their colonization on the host roots.

Soil organic matter was negatively correlated with the RAs of three main bacterial taxa: phylum Gemmatimonadetes and its members Holophagae (belonging to Acidobacteria) and *Ohtaekwangia* (belonging to Bacteroidetes) (Figure 6H). However, pH was negatively correlated with the RAs of many members of Bacteroidetes and Spartobacteria of Verrucomicrobia and was positively correlated with the RA of Rhodospirillales of Proteobacteria (Figure 6I). Verrucomicrobial elevational distribution was strongly influenced by soil pH and carbon/nitrogen ratio (Shen et al., 2017). Malik et al. (2018) discovered distinct pH controls of microbial mechanisms of carbon accumulation. This finding indicated that the combined effect of SOM and pH shaped the soil decomposition pattern of the dominant bacteria (Hernández et al., 2015; Malik et al., 2018; Martínez-García et al., 2018; Wan et al., 2020).

Additionally, for the fungal community, SOM was positively correlated with fungal alpha-diversity (Chao) and negatively correlated with the RAs of most members of the phylum Ascomycota, whereas pH was positively correlated with the RAs of some members of Zygomycota. These results are in line with the finding that SOM was more important than pH in inhibiting the dominant fungi involved in degrading organic materials and in inhibiting pathogen growth (Tian et al., 2016). Furthermore, SOM was positively correlated with the RAs of some functional groups related to the C cycle and negatively correlated with the RAs of ligninolysis, fumarate respiration, nitrite ammonification, and nitrate ammonification groups of the bacterial community and pathotrophic groups of fungi, whereas pH was not correlated with the RAs of functional groups (Figure 6). These results suggested that SOM was the main driver of microbial functional groups. Changes in SOM and pH mediating the microbial community were associated with increases in saccharide and OA contents and decreases in OHC and alcohol compounds (Tian et al., 2016; Cui et al., 2018; Li et al., 2018).

## Conclusion

In conclusion, we found that root exudates from aromatic plants shaped the diversity, structure, composition and function of the soil microbial community, which regulated C and N nutrients during SOM decomposition. Intercropping significantly reduced the RAs of saprotrophic and pathotrophic groups. The TN and AN contents, species richness alpha-diversity of bacterial community, and the symbiotic group of the fungal community were higher in Tr1 and Tr2 than in Tr3. We suggest that intercropping with basil and summer savory is more beneficial to pear orchards than intercropping with blue mink. However, the biomass of the intercrops was not determined in this study. To identify the main beneficial factors in intercropping systems, the contents of root exudates and the biomass of intercrops both need to be studied.

## Data Availability Statement

The data presented in the study are deposited in the NCBI repository, accession number (PRJNA685959).

## Author Contributions

MH, MS, JT, BS, and YH contributed to the data curation. YY contributed to the funding acquisition. YZ, MH, and MS contributed to the investigation. YZ and JZ contributed to the writing (original draft). YY contributed to the writing (review and editing). All authors contributed to the article and approved the submitted version.

## Conflict of Interest

The authors declare that the research was conducted in the absence of any commercial or financial relationships that could be construed as a potential conflict of interest.

## Footnotes

1 https://mothur.org

2 https://rdp.cme.msu.edu/

3 www.zoology.ubc.ca/louca/FAPROTAX

4 http://www.stbates.org/guilds/app.php

5 v3.5.1, https://www.r-project.org
