# Supplementary material for: Intercropping With Aromatic Plants Increased the Soil Organic Matter Content and Changed the Microbial Community in a Pear Orchard
Source: Front Microbiol. 2021 Feb 12;12:616932. doi: 10.3389/fmicb.2021.616932 (PMC7907656; doi:10.3389/fmicb.2021.616932)
Supplement: Supplementary file 1 [file Data_Sheet_1.doc]

**Supplementary materials for on-line publication only**

**Supplementary Fig. S1.** Schematics of the intercropping of different aromatic plants in the field experiment. (a) Schematic of three models with four treatments; (b) Schematic of each plot; (c) Schematic of five sampling points in each plot. The different treatments were separated from adjacent plots by a 10 m isolation belt. CK (Model-1), clean tillage with only the mature pear trees; Model-2 included two species intercropped with Lamiaceae plant, i.e., basil (*Ocimum basilicum* L. Tr1); summer savory (*Satureja hortensis* L. Tr2); Model-3 intercropped with Asteraceae (*Ageratum houstonianum* Mill. Tr3).

**Supplementary Fig. S2.** The heatmap of clustering and relative percentages of treatments with respect to CK according the relative abundance of dominant microbes (≥ 1%) at the phylum, class, order, family and genus levels. (a) bacterial communities; (b) fungal communities. Different letters indicate significant differences (*P* < 0.05) based on Duncan’s multiple range test. Relative percentages of treatments with respect to CK represent differences between the relative abundance of dominant microbes in the treatments and CK divided by the relative abundance of dominant microbes in CK.

**Supplementary Table S1** The total bacterial community composition at the phylum, class, order, family and genus levels in soil with different intercropping models.

| **Taxa** | **Name** | **CK** |  | **Tr1** | **Tr2** |  | **Tr3** |
| --- | --- | --- | --- | --- | --- | --- | --- |
| **Phylum** | **Proteobacteria** | 26.5±1.48ab |  | 26.05 ± 1.47ab | 28.98 ± 0.43a |  | 23.59 ± 2.53b |
| **Class** | **Alphaproteobacteria** | 9.82±0.4ab |  | 8.93 ± 0.73b | 9.96 ± 0.54ab |  | 10.72 ± 0.92a |
|  | **Betaproteobacteria** | 7.08 ± 0.9a |  | 7.18 ± 0.68a | 7.71 ± 0.51a |  | 6.02± 1.37a |
|  | **Deltaproteobacteria** | 5.29 ± 0.79a |  | 5.24 ± 0.77a | 5.35 ± 0.34a |  | 3.02 ± 0.23b |
|  | **Gammaproteobacteria** | 3.59 ± 0.02b |  | 3.98± 0.97ab | 4.96 ± 0.22a |  | 3.42 ± 0.88b |
| **Order** | **Sphingomonadales** | 3.98 ± 0.25ab |  | 3.8 ± 1.17ab | 2.67 ± 0.17b |  | 4.95 ± 0.91a |
|  | **Myxococcales** | 3.85 ± 0.79a |  | 3.6 ± 0.51a | 3.49 ± 0.26a |  | 2 ± 0.14b |
|  | **Rhizobiales** | 2.33 ± 0.33a |  | 2.45 ± 0.56a | 2.79 ± 0.18a |  | 3.02 ± 0.28a |
|  | **Xanthomonadales** | 2.22 ± 0.06a |  | 2.42 ± 0.31a | 2.32 ± 0.09a |  | 2.47 ± 0.79a |
|  | **Burkholderiales** | 1.74 ± 0.10b |  | 2.37 ± 0.29ab | 1.86 ± 0.29b |  | 2.55 ± 0.55a |
|  | **Rhodospirillales** | 1.73 ± 0.27ab |  | 1.29 ± 0.2b | 2.15 ± 0.37a |  | 1.24 ± 0.16b |
| **Family** | **Sphingomonadacea** | 3.33 ± 0.21ab |  | 3.11 ± 1.02ab | 2.28 ± 0.18b |  | 4.2 ± 0.72a |
|  | **Comamonadaceae** | 1.29 ± 0.15a |  | 1.6 ± 0.23a | 1.34 ± 0.36a |  | 1.67 ± 0.37a |
|  | **Xanthomonadaceae** | 1.36 ± 0.17a |  | 1.6 ± 0.42a | 1.13 ± 0.07a |  | 2.02 ± 0.8a |
|  | **Haliangiaceae** | 1.1 ± 0.19a |  | 0.94 ± 0.11a | 0.87 ± 0.09a |  | 0.47 ± 0.08b |
| **Genus** | ***Sphingomonas*** | 2.97 ± 0.23a |  | 2.56 ± 0.93a | 1.92 ± 0.16a |  | 3.07 ± 0.66a |
| **Phylum** | **Acidobacteria** | 14.09 ± 2.02a |  | 16.38 ± 1.41a | 13.96 ± 1.88a |  | 13.83 ± 1.73a |
| **Class** | **Acidobacteria** | 11.43 ± 1.57a |  | 14.51 ± 1.25a | 12.57 ± 1.9a |  | 12.18 ± 1.89a |
|  | **Holophagae** | 2.4 ± 0.4a |  | 1.52 ± 0.31b | 1.09 ± 0.16b |  | 1.36 ± 0.27b |
| **Order** | **Subgroup 6** | 6.76 ± 0.82a |  | 7.14 ± 0.82a | 6.78 ± 0.91a |  | 5.79 ± 0.17a |
|  | **Subgroup 4** | 3.08 ± 0.47b |  | 5.88 ± 0.39a | 3.87 ± 1.34ab |  | 5.18 ± 1.98ab |
|  | **Subgroup 7** | 2.39 ± 0.4a |  | 1.51 ± 0.32b | 1.07 ± 0.15b |  | 1.35 ± 0.26b |
| **Family** | **RB41** | 1.75 ± 0.31a |  | 3.13 ± 0.26a | 2.18 ± 0.8a |  | 2.85 ± 1.08a |
| **Genus** | ***Blastocatella*** | 1.16 ± 0.12b |  | 2.22 ± 0.43a | 1.23 ± 0.37b |  | 2.01 ± 0.72ab |
| **Phylum** | **Actinobacteria** | 9.08 ± 0.97b |  | 7.69 ± 2.03b | 7.25 ± 2.51b |  | 16.3 ± 2.53a |
| **Class** | **Actinobacteri** | 3.7 ± 0.28b |  | 2.99 ± 1.12b | 3.24 ± 1.29b |  | 7.03 ± 0.98a |
|  | **Acidimicrobii** | 2.01 ± 0.47b |  | 1.86 ± 0.42b | 1.4 ± 0.3b |  | 3.4 ± 0.86a |
|  | **Thermoleophilia** | 2.09 ± 0.29b |  | 1.5 ± 0.36b | 1.6 ± 0.71b |  | 3.31 ± 0.67a |
| **Order** | **Acidimicrobiale** | 2.01 ± 0.47b |  | 1.86 ± 0.42b | 1.4 ± 0.3b |  | 3.4 ± 0.86a |
|  | **Micrococcales** | 0.99 ± 0.05b |  | 1.1 ± 0.56b | 1 ± 0.17b |  | 2.71 ± 0.18a |
|  | **Propionibacteriales** | 1.09 ± 0.14b |  | 0.76 ± 0.25b | 0.8 ± 0.37b |  | 2.08 ± 0.68a |
|  | **Solirubrobacterales** | 1.02 ± 0.11b |  | 0.82 ± 0.25b | 0.72 ± 0.23b |  | 1.96 ± 0.32a |
| **Family** | **Micrococcaceae** | 0.66 ± 0.03b |  | 0.86 ± 0.51b | 0.72 ± 0.08b |  | 2.07 ± 0.16a |
|  | **Nocardioidaceae** | 1.07 ± 0.14b |  | 0.73 ± 0.24b | 0.78 ± 0.37b |  | 2.04 ± 0.69a |
|  | **Gaiellaceae** | 1.02 ± 0.19a |  | 0.63 ± 0.1a | 0.84 ± 0.5a |  | 1.26 ± 0.39a |
| **Genus** | ***Gaiella*** | 1.02 ± 0.19a |  | 0.63 ± 0.1a | 0.84 ± 0.5a |  | 1.26 ± 0.39a |
| **Phylum** | **Bacteroidetes** | 8.43 ± 1.19b |  | 10.23 ± 0.82a | 8.72 ± 0.45ab |  | 8.47 ± 0.69b |
| **Class** | **Sphingobacteriia** | 3.94 ± 0.67b |  | 5.37 ± 1a | 3.31 ± 0.1b |  | 5.14 ± 0.26a |
|  | **Cytophagia** | 3.13 ± 0.32ab |  | 2.63 ± 0.08b | 3.22 ± 0.07a |  | 1.71 ± 0.42c |
| **Order** | **Sphingobacteriales** | 3.94 ± 0.67b |  | 5.37 ± 1a | 3.31 ± 0.1b |  | 5.14 ± 0.26a |
|  | **Cytophagales** | 3.05 ± 0.31ab |  | 2.6 ± 0.09b | 3.18 ± 0.08a |  | 1.69 ± 0.42c |
| **Family** | **Chitinophagaceae** | 2.41 ± 0.43bc |  | 3.11 ± 0.81ab | 2.06 ± 0.17c |  | 3.8 ± 0.2a |
|  | **env.OPS 17** | 0.96 ± 0.22b |  | 1.56 ± 0.5a | 0.65 ± 0.16b |  | 0.58 ± 0.16b |
|  | **Cytophagaceae** | 2.86 ± 0.39a |  | 2.51 ± 0.11a | 3.02 ± 0.03a |  | 1.58 ± 0.36b |
| **Genus** | ***Ohtaekwangia*** | 2.03 ± 0.52a |  | 1.28 ± 0.1bc | 1.64 ± 0.3ab |  | 0.94 ± 0.31c |
| **Phylum** | **Planctomycetes** | 7.64 ± 0.93a |  | 7.95 ± 0.1a | 7.52 ± 0.86a |  | 7.41 ± 2.64a |
| **Class** | **Phycisphaerae** | 4.38 ± 0.64a |  | 4.75 ± 0.72a | 3.83 ± 0.89a |  | 4.15 ± 1.89a |
|  | **Planctomycetacia** | 2.76 ± 0.35a |  | 2.68 ± 0.63a | 2.89 ± 0.27a |  | 3 ± 0.76a |
| **Order** | **WD2101 soil group** | 4.04 ± 0.62a |  | 4.41 ± 0.79a | 3.38 ± 0.75a |  | 3.93 ± 1.82a |
|  | **Planctomycetales** | 2.76 ± 0.35a |  | 2.68 ± 0.63a | 2.89 ± 0.27a |  | 3 ± 0.76a |
| **Family** | **Planctomycetaceae** | 2.76 ± 0.35a |  | 2.68 ± 0.63a | 2.89 ± 0.27a |  | 3 ±0.76a |
| **Phylum** | **Verrucomicrobia** | 5.29 ± 0.74b |  | 6.44 ± 0.1a | 5.16 ± 0.5b |  | 5.93 ± 0.65ab |
| **Class** | **OPB35 soil group** | 3.32 ± 0.42a |  | 3.57 ± 0.37a | 2.94 ± 0.15a |  | 2.85 ± 0.46a |
|  | **Spartobacteria** | 0.53 ± 0.12b |  | 1.03 ± 0.22a | 0.57 ± 0.27b |  | 1.11 ± 0.3a |
|  | **Verrucomicrobiae** | 0.38 ± 0.15c |  | 0.78 ± 0.14b | 0.49 ± 0.1bc |  | 1.09 ± 0.22a |
| **Order** | **Verrucomicrobiales** | 0.38 ± 0.15c |  | 0.78 ± 0.14b | 0.49 ± 0.1bc |  | 1.08 ± 0.22a |
| **Family** | **Verrucomicrobiaceae** | 0.36 ± 0.14b |  | 0.76 ± 0.14a | 0.46 ± 0.09b |  | 1.05 ± 0.22a |
| **Phylum** | **Gemmatimonadetes** | 6.35 ± 0.39a |  | 5.72 ± 0.78ab | 5.1 ± 0.33bc |  | 4.08 ± 0.8c |
| **Class** | **Gemmatimonadetes** | 6.35 ± 0.39a |  | 5.72 ± 0.78ab | 5.1 ± 0.33bc |  | 4.08 ± 0.8c |
| **Order** | **Gemmatimonadales** | 4.41 ± 0.38a |  | 3.96 ± 0.49a | 3.61 ± 0.37a |  | 3.49 ± 0.62a |
|  | **S0134 terrestrial group** | 1.52 ± 0.05a |  | 1.37 ± 0.38ab | 1.01 ± 0.08b |  | 0.5 ± 0.21c |
| **Family** | **Gemmatimonadaceae** | 4.41 ± 0.38a |  | 3.96 ± 0.49a | 3.61 ± 0.37a |  | 3.49 ± 0.62a |
| **Genus** | ***Gemmatimonas*** | 3.07 ± 0.27a |  | 2.73 ± 0.32ab | 2.46 ± 0.3ab |  | 2.38 ± 0.36b |
| **Phylum** | **Chloroflexi** | 3.76 ± 0.39ab |  | 2.8 ± 0.35b | 3.58 ± 0.88ab |  | 4.41 ± 0.19a |
| **Phylum** | **Cyanobacteria** | 1.09 ± 0.36b |  | 1.4 ± 0.21b | 1.32 ± 0.77b |  | 3.21 ± 0.07a |
| **Class** | **Cyanobacteria** | 0.47 ± 0.25b |  | 0.61 ± 0.19b | 0.29 ± 0.2b |  | 1.47 ± 0.1a |

Note: Values are the mean ± SD (n = 3). Different letters indicate significant differences (*P* < 0.05) for the individual factors based on one-way ANOVA followed by Duncan’s multiple range test.

**Supplementary Table S2** The total fungal community composition at the phylum, class, order, family and genus levels in soil with different intercropping models.

| **Taxa** | **Name** | CK |  | Tr | Tr2 |  | Tr3 |
| --- | --- | --- | --- | --- | --- | --- | --- |
| **Phylum** | **Ascomycota** | 57.26 ± 12.5a |  | 28.7 ± 1.02bc | 42.18 ± 4.77b |  | 23.64 ± 7.68c |
| **Class** | **Sordariomycetes** | 33.36 ± 6.83a |  | 17.37 ± 1.99b | 27.6 ± 4.66a |  | 12.18 ± 3.93b |
|  | **Pezizomycetes** | 9.51 ± 11.08a |  | 2.68 ± 2.04a | 3.73 ± 3.94a |  | 2.26 ± 0.26a |
|  | **AscomycotaIncertaesedis** | 6.89 ± 6.33a |  | 1.09 ± 0.12a | 0.78 ± 0.24a |  | 1.41 ± 0.81a |
|  | **Dothideomycetes** | 3.72 ± 1.03a |  | 3.44 ± 0.39a | 4.28 ± 1.66a |  | 5.3 ± 2.43a |
|  | **Leotiomycetes** | 0.26 ± 0.3a |  | 1.01 ± 0.35a | 1.03 ± 0.64a |  | 0.63 ± 0.38a |
|  | **Eurotiomycetes** | 0.88 ± 0.39a |  | 0.88 ± 0.35a | 1.26 ± 1.02a |  | 0.29 ± 0.2a |
| **Order** | **Sordariales** | 21.52 ± 8.63a |  | 6.35 ± 0.9bc | 15.02 ± 3.79ab |  | 4.43 ± 1.76c |
|  | **Hypocreales** | 9.42 ± 6.73a |  | 6.95 ± 1.7a | 8.66 ± 1.67a |  | 4.27 ± 0.88a |
|  | **Xylariales** | 0.06 ± 0.03b |  | 1.56 ± 0.28a | 0.43 ± 0.16b |  | 1.74 ± 1.01a |
|  | **Ascomycota order Incertae sedis** | 6.89 ± 6.33a |  | 1.09 ± 0.12a | 0.78 ± 0.24a |  | 1.41 ± 0.81a |
|  | **Pleosporales** | 3.61 ± 1.07a |  | 2.84 ± 0.22a | 2.95 ± 0.79a |  | 3.25 ± 0.99a |
|  | **Pezizales** | 9.36 ± 10.83a |  | 2.67 ± 2.04a | 3.55 ± 3.64a |  | 2.24 ± 0.23a |
| **Family** | **Ascomycota family Incertae sedis** | 6.89 ± 6.33a |  | 1.09 ± 0.12a | 0.78 ± 0.24a |  | 1.41 ± 0.81a |
|  | **Pleosporales family Incertae sedis** | 0.77 ± 0.33a |  | 1.17 ± 0.6a | 1.23 ± 0.39a |  | 1.32 ± 0.4a |
|  | **Chaetomiaceae** | 17.03 ± 9.1a |  | 2.57 ± 0.64b | 11.19 ± 4.48ab |  | 2.63 ± 2b |
|  | **Nectriaceae** | 7.11 ± 5.76a |  | 4.59 ± 2.48a | 6.05 ± 1.1a |  | 2.99 ± 0.63a |
|  | **Ascobolaceae** | 8.3 ± 10.01a |  | 0.89 ± 0.56a | 2.84 ± 3.67a |  | 1.89 ± 0.18a |
|  | **Lasiosphaeriaceae** | 2.37 ± 1.57a |  | 2.26 ± 0.92a | 2.21 ± 0.91a |  | 0.94 ± 0.73a |
|  | **Hypocreales family Incertae sedis** | 2.09 ± 1.61a |  | 1.54 ± 0.29a | 1.48 ± 0.66a |  | 1.01 ± 0.29a |
| **Genus** | ***Ciliophora*** | 6.49 ± 0.87a |  | 0.65 ± 0.17b | 0.28 ± 0.02b |  | 0.66 ± 0.19b |
|  | ***Myrothecium*** | 1.69 ± 0.07a |  | 0.99 ± 0.1b | 0.79 ± 0.04c |  | 0.47 ± 0.01d |
|  | ***Gibberella*** | 6.44 ± 0.01a |  | 3.05 ± 0.05c | 4.23 ± 0.02b |  | 2.37 ± 0.01d |
|  | ***Trichocladium*** | 1.03±0.02a |  | 0.38 ± 0.02c | 0.98 ± 0.02b |  | 0.26 ± 0d |
|  | ***Podospora*** | 1.16±0.01a |  | 0.55 ± 0.02b | 0.37 ± 0.01c |  | 0.2 ± 0.01d |
|  | ***Monographella*** | 0.04±0.01b |  | 0.77 ± 0.08ab | 0.39 ± 0.14b |  | 1.52 ± 0.87a |
| **Phylum** | **Basidiomycota** | 2.51 ± 1.47b |  | 18.17 ± 7.34a | 13.53 ± 8.66ab |  | 4.78 ± 2.74b |
| **Class** | **Tremellomycetes** | 0.27 ± 0.23b |  | 13.6 ± 8.16a | 10.41 ± 7.46ab |  | 2.34 ± 1.78b |
|  | **Agaricomycetes** | 2.14 ± 1.29a |  | 3.85 ± 2.23a | 2.6 ± 1.87a |  | 1.48 ± 0.81a |
| **Order** | **Cystofilobasidiales** | 0.11 ± 0.12b |  | 12.44 ± 7.94a | 9.44 ± 6.9ab |  | 1.33 ± 1.77b |
|  | **Agaricales** | 1.36 ± 1.17a |  | 2.63 ± 2.15a | 0.59 ± 0.52a |  | 0.8 ± 0.68a |
| **Family** | **Cystofilobasidiaceae** | 0.11 ± 0.12b |  | 12.44 ± 7.94a | 9.44 ± 6.89ab |  | 1.33 ± 1.77b |
| **Genus** | ***Entoloma*** | 0.59 ± 0.08a |  | 1.47 ± 2.48a | 0.21 ± 0.34a |  | 0.03 ± 0.02a |
|  | ***Guehomyces*** | 0.09 ± 0.1b |  | 12.38 ± 7.86a | 9.42 ± 6.89ab |  | 1.3 ± 1.79b |
| **Phylum** | **Chytridiomycota** | 0.84 ± 0.35b |  | 2.56 ± 1.38a | 0.52 ± 0.13b |  | 0.66 ± 0.33b |
| **Class** | **Chytridiomycetes** | 0.28 ± 0.12b |  | 1.84 ± 1.03a | 0.27 ± 0.17b |  | 0.54 ± 0.28b |
| **Phylum** | **Glomeromycota** | 1.33 ± 1.55a |  | 4.37 ± 3.47a | 2.35 ± 0.83a |  | 0.76 ±0.35a |
| **Class** | **Glomeromycetes** | 1.31 ± 1.52a |  | 4.24 ± 3.34a | 2.33 ± 0.81a |  | 0.74 ± 0.35a |
| **Order** | **Glomerales** | 1.3 ± 1.52a |  | 4.19 ± 3.31a | 2.02 ± 0.53a |  | 0.71 ± 0.33a |
| **Family** | **Glomeraceae** | 1.27 ± 1.54a |  | 2.35 ± 2.02a | 1.46 ± 0.56a |  | 0.44 ± 0.14a |
| **Genus** | ***Glomus*** | 0.18 ± 0.1b |  | 1.02 ± 0.8a | 0.41 ± 0.06ab |  | 0.31 ± 0.15ab |
| **Phylum** | **Zygomycota** | 5.37 ± 3.75a |  | 4.64 ± 1.8a | 5.09 ± 2.99a |  | 3.43 ± 1.56a |
| **Class** | **Zygomycota-classIncertaesedis** | 5.37 ± 3.75a |  | 4.64 ± 1.8a | 5.09 ± 2.99a |  | 3.43 ± 1.56a |
| **Order** | **Mortierellales** | 5.34 ± 3.78a |  | 4.33 ± 1.74a | 4.93 ± 3.07a |  | 3.37 ± 1.54a |
| **Family** | **Mortierellaceae** | 5.34 ± 3.78a |  | 4.33 ± 1.74a | 4.93 ± 3.07a |  | 3.37 ± 1.54a |
| **Genus** | ***Mortierella*** | 5.34 ± 0.37a |  | 4.33 ± 0.31ab | 4.93 ± 1.16a |  | 3.37 ± 0.55b |
| **Phylum** | **Ciliophora** | 1.44 ± 0.18a |  | 0.99 ± 0.29ab | 0.48 ± 0.21c |  | 0.92 ± 0.31bc |

Note: Values are the mean ± SD (n = 3). Different letters indicate significant differences (*P* < 0.05) for the individual factors based on one-way ANOVA followed by Duncan’s multiple range test.


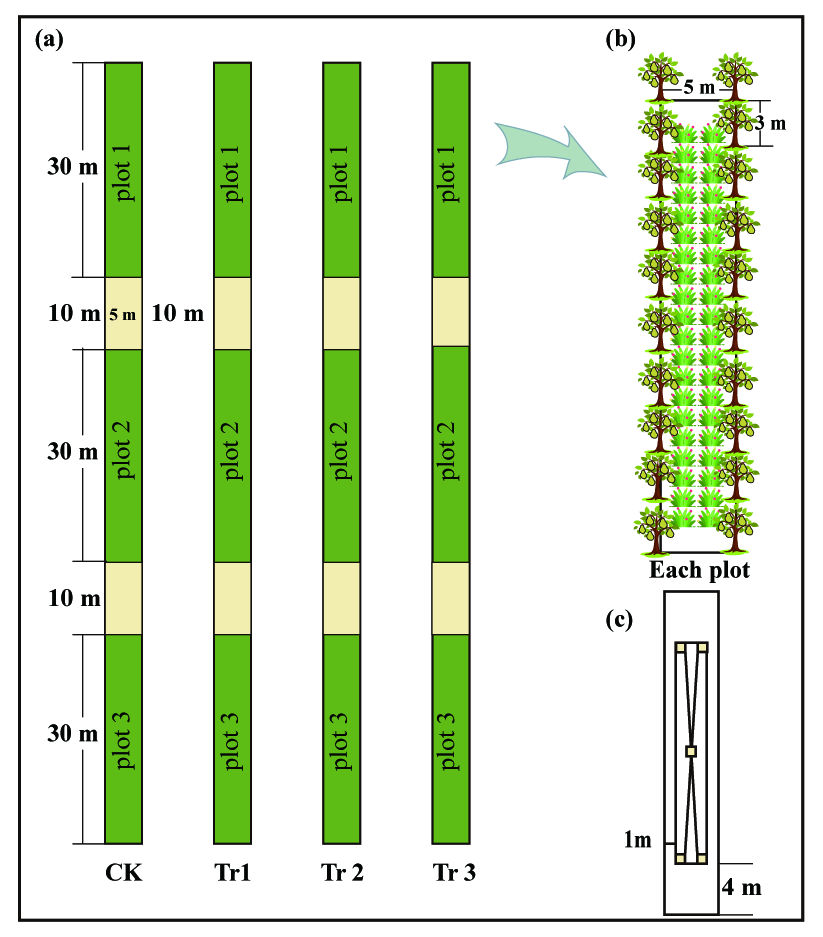


**Supplementary Fig. S1.**


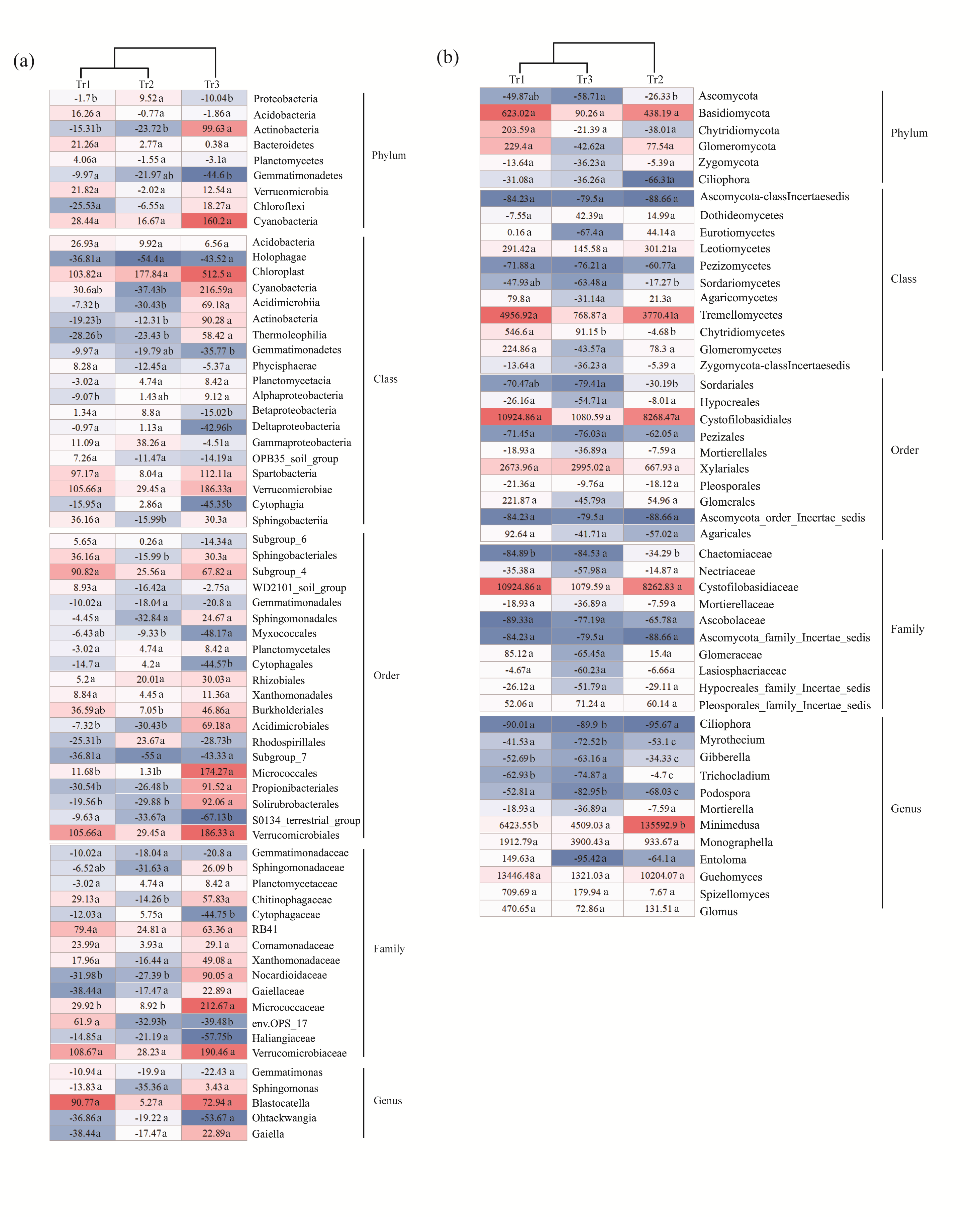


**Supplementary Fig. S2.**
